# The Advent of Salivary Breast Cancer Biomarker Detection Using Affinity Sensors

**DOI:** 10.3390/s19102373

**Published:** 2019-05-23

**Authors:** Imad Abrao Nemeir, Joseph Saab, Walid Hleihel, Abdelhamid Errachid, Nicole Jafferzic-Renault, Nadia Zine

**Affiliations:** 1 Faculty of Sciences, Holy Spirit University of Kaslik, 446 Jounieh, Mount Lebanon, Lebanon; josephsaab@usek.edu.lb (J.S.); walidhleihel@usek.edu.lb (W.H.); 2 Institut des Sciences Analytiques, Université de Lyon, Claude Bernard Lyon 1, UMR 5280, CNRS – 5, rue de la Doua, 69100 Villeurbanne, France; abdelhamid.errachid-el-salhi@univ-lyon1.fr (A.E.); nicole.jaffrezic@univ-lyon1.fr (N.J.-R.)

**Keywords:** Breast Cancer (BC), Diagnosis, affinity biosensors (AS), enzyme-linked immunosorbent assay (ELISA)

## Abstract

Breast Cancer is one of the world’s most notorious diseases affecting two million women in 2018 worldwide. It is a highly heterogeneous disease, making it difficult to treat. However, its linear progression makes it a candidate for early screening programs, and the earlier its detection the higher the chance of recovery. However, one key hurdle for breast cancer screening is the fact that most screening techniques are expensive, time-consuming, and cumbersome, making them impractical for use in several parts of the world. One current trend in breast cancer detection has pointed to a possible solution, the use of salivary breast cancer biomarkers. Saliva is an attractive medium for diagnosis because it is readily available in large quantities, easy to obtain at low cost, and contains all the biomarkers present in blood, albeit in lower quantities. Affinity sensors are devices that detect molecules through their interactions with biological recognition molecules. Their low cost, high sensitivity, and selectivity, as well as rapid detection time make them an attractive alternative to traditional means of detection. In this review article, we discuss the current status of breast cancer diagnosis, its salivary biomarkers, as well as the current trends in the development of affinity sensors for their detection.

## 1. Introduction

In the latest worldwide study about cancer, Breast Cancer (BC) took center-stage as the most frequently diagnosed type of cancer in the world, accounting on its own for 25% of all cases and 15% of all cancer related deaths [1]. However, due to the fact of the small preclinical tumors’ tendency for slow metastasis, screening tests are a valid strategy for early detection [2]. Many detection techniques have also been developed for that purpose; from mammography as imaging technique [3], immunohistochemistry (IHC) for biomarker detection [4], enzyme linked immunosorbent assays for blood analysis (ELISA) [5]. However, mammography, being the golden standard for early BC detection, has been fraught with sensitivity issues [6] while clinical breast cancer screening and self-breast cancer screening have major biases and generally lead to false positives [7].

On the other hand, biological molecules-based techniques such as IHC have played a major role in satisfying the needs for BC diagnostics. However, and despite their efficiency, these techniques possess their own limitations making them less than ideal for screening purposes [8]; in addition to their expense and long run time, the need for an invasive technique such as biopsy in order to obtain a sample discourages their use [4].

Hence, why did the development of affinity sensors as an alternative come to be?—affinity sensors are sensitive, cheap, and provide a quick result. They are unique devices that turn biological interactions into a physical signal that can be both qualified and quantified at low concentrations, which has opened the door for them to be used in the detection of biomarkers in biological fluids other than blood [9].

Saliva is one such fluid. Secreted in the oral cavity as an aid for digestion, current research has shown that, in addition to its role in digestion, it contains a large host of biomarkers covering different diseases (including BC) and ailments of the body, thus prompting some to call it “The mirror of the body” [10]. A leading objective for any healthcare research is to develop a tool that is both effective as well as noninvasive. In this regard, saliva fits the above criteria perfectly; as a complex oral fluid, it can be collected noninvasively, all the while possessing considerable potential for detecting and surveilling general health and disease [11].

In this review article, we explore BC’s epidemiology in Europe and the Middle East, followed by a brief overview of its biology and medical classifications as well as the biomarkers that are used for these classifications. Afterwards, we highlight the recent advancements made in the field of affinity sensors and their use for the detection of the aforementioned biomarkers.

## 2. Prevalence of Breast Cancer Internationally, Regionally, and Locally

As mentioned earlier, BC is the most commonly diagnosed and leading cause of cancer death among females worldwide, with about two million new cases and 626,700 deaths in 2018, it singlehandedly accounts for 25% of all cancer cases and 15% of all cancer deaths among the female population [1,12].

**Figure 1:**
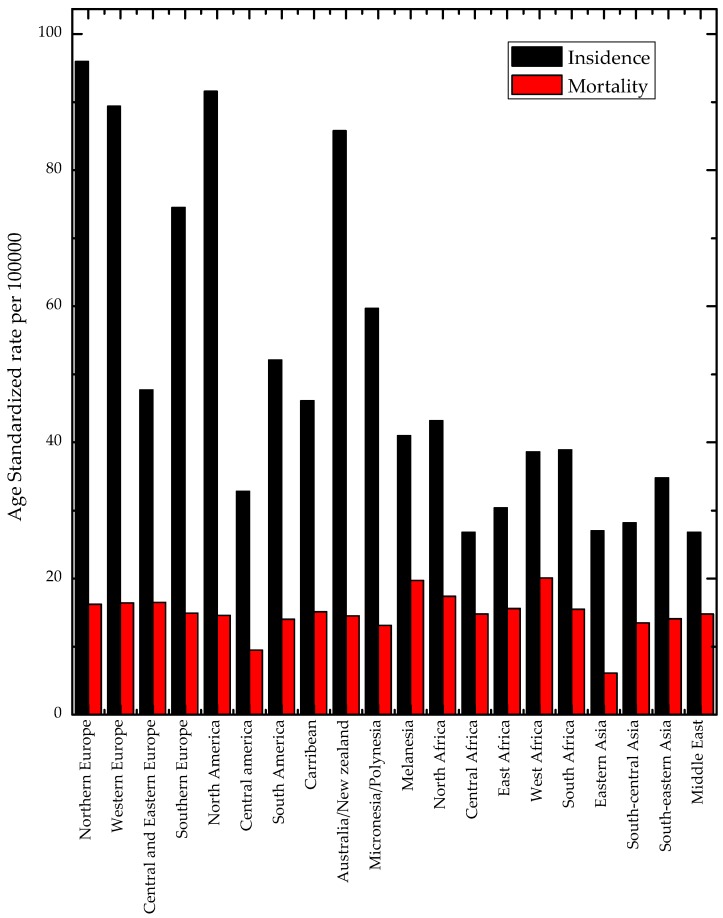
Breast cancer incidence and mortality rates by world area [1].

While developed countries account for about one-half of all breast cancer cases and 38% of deaths, other areas are not so affected with the Caribbean and Latin America having less incidents than the developed world, and Asia and Africa having the least amount of BC cases. These differences can be seen in Figure 1.

International variations in breast cancer incidence are due to differing factors from socio-economics (the availability of early detection, recent use of oral contraceptives, and never having children) as well as biological factors (hormones and hormonal therapy, obesity, physical activity, and alcohol consumption) [1,12,13].

### Regional and National Characteristics of Breast Cancer

In relations to this review, only two regions interest us particularly, Western Europe and the Arab world (and in particular, France and Lebanon).

Being the leading cancer site in women in all countries of Europe in 2012 (Figure 1), BC has a 3-fold variation (49–148 per 100,000) with a clear geographical pattern. High incidence rates were estimated in Western and Northern European countries. In comparison, incidence rates in Eastern European countries were much lower. The range of mortality rates also varies along the same lines [14]. 

The same can also be said of the Arab world, where despite incidents being generally lower than that of Europe, they are steadily increasing and are expected to match European numbers eventually although the true prevalence of breast cancer in Arab populations is uncertain. Recent findings suggest that BC might be more common than expected, highlighting the importance of further research to assess the actual prevalence of BC in Arab countries.

**Figure 2:**
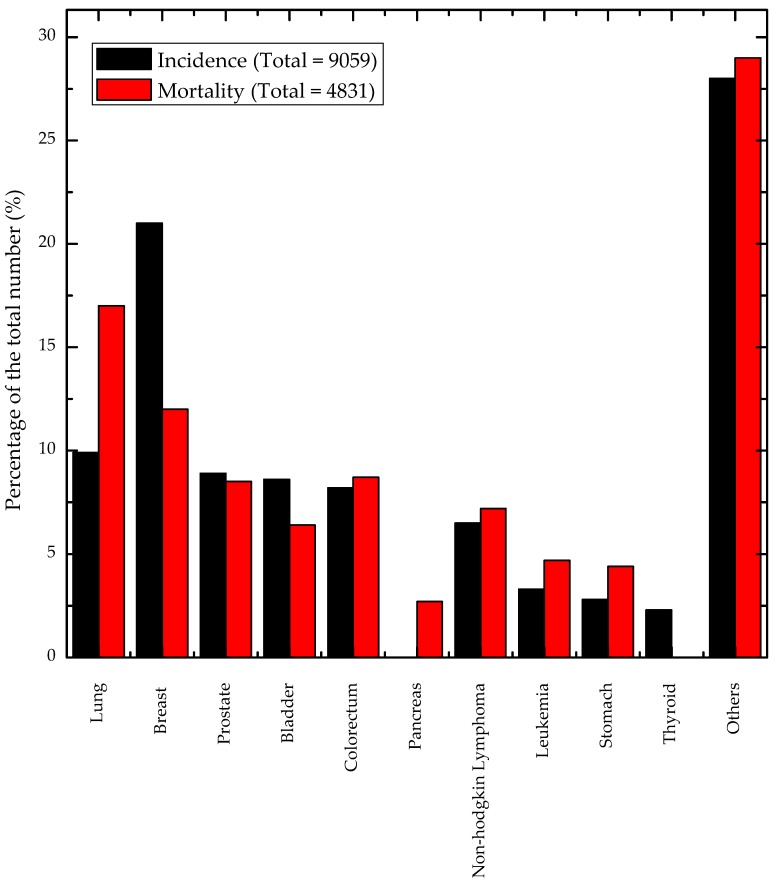
Rates and percentage of incidence and mortality of cancer in Lebanon [15].

A key point to make is that according to the International Agency for Research on Cancer (IARC) Globocan 2012 report, BC incidence is the largest site for cancer in Lebanon, accounting for 21% of all cancer incidence in 2012, but only accounting for 12% of the mortality rate, second after lung cancer (17%). Full statistics on cancer distribution in Lebanon can be found in Figure 2. The numbers in the metropolitan area of France differ slightly; BC has the second largest number of incidence at 14%, while only being the third in terms of mortality accounting for 7.7% of all deaths after colorectal cancer (11%) and lung cancer (20%) [1]. The reason for this discrepancy between high levels of incidence and lower levels of mortality will be further discussed later.

Arabs have differing clinicopathological features of BC from Europeans, an important note is the average age at presentation which is almost a decade younger. Even more so is the difference in immunohistochemical profile of the molecular classes which suggests that there are clinicopathological differences in addition to the discrepancies in the expression profiling in both regions [16].

These differences can be attributed to the accessibility in Western countries of well-established BC screening approaches and useful public awareness programs as it has been repeatedly shown that Arab women with early, but asymptomatic, BC fail to seek medical consultation in the early stages. Therefore, their tumors tend to be larger, with greater histologic grades and increased lymph node involvement.

## 3. Biological Characteristics of Breast Cancer

BC is highly diverse in its etiology and pathological features; in some cases, it shows slow growth with excellent prognosis, whereas other cases are highly aggressive. This difference can be divided into three classes; tumor grade, morphological classification, and molecular classification [17].

**Figure 3:**
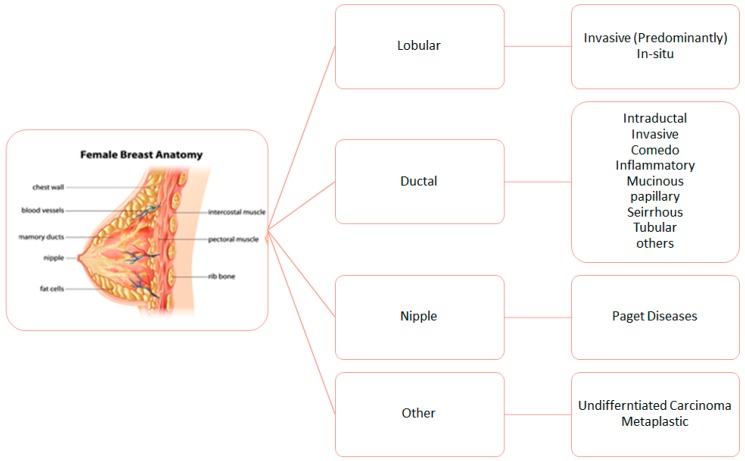
Diseases of the breast tissue and their origins [18].

The most common definition of grade, also known as Nottingham combined histologic grade, is based on the semi-quantitative evaluation of morphologic features related to tumor differentiation from normal cells. BC has two main morphological umbrellas and they are its invasiveness (invasive or in situ carcinoma), and its place of origin (ductal and lobular carcinoma). It is worth noting that both these tumors come from the same parts of the breast, the terminal duct lobular unit (TDLU). A schematic of the location of pre-cancer type can be found in Figure 3. This grading index, also known as the Nottingham Prognostic Index (NPI) is an important tool for prognostics. However, the biggest issue with the grade system is its reproducibility [18].

With regard to its molecular classification, again, BC has been divided into two major subtypes, the estrogen receptor positive (ER+) and estrogen receptor negative (ER−) subtypes; these two subtypes can be further divided into several subtypes found in Table 1.

In addition to these subtypes, two BC terms also come up often, and they are triple negative and inflammatory BC.

Triple negative subtypes are an umbrella term used to identify types of BC that show no immunohistochemical traces of ER, PR, and HER2, hence the term “triple negative” [20].

Inflammatory BC, an aggressive form of BC due to its strong metastatic potential, is diagnosed based on clinical signs of inflammation (redness, edema, “peau d’orange”) coming from and encompassing more than one-third of the breast [21].

The relative subjectivity of the clinical symptoms, and the occasional diagnosis using only pathological criteria is the cause of extreme heterogeneity throughout the affected population across different clinical and scientific reports, hindering any researchers working on it [22,23]. 

As for the genomic diversity of BC, several genetic factors have been found to make an individual predisposed to BC. BRCA1 and BRCA2 are the most notable ones [24]. Other genes also exist, such as MYC [25], ATM [26], CDH1 [27], CHEK2 [28], and p53 just to name a few [29], but listing all of them here is beyond the scope of this review. It is worth noting that several assays have been developed for the detection of these genes and their classification such as Oncotype DX and MammaPrint [23].

### Regional Immunohistochemical Characteristics of BC

Estrogen-receptor positive BC accounts for 70% of Western women whilst overall Arab women present a majority in estrogen-receptor negative BC with a large prevalence of triple negative BC (20% in the UAE to 39% in Saudi women) with Lebanese women being an exception (9.3%), with rates comparable as to that of European (9%) women. In addition, HER2 overexpression is much more prevalent in the Arab region (39.5% in Lebanon) than in other parts of the world [16,30,31].

## 4. Early Detection of Breast Cancer Biomarkers Diagnosis Tests

**Figure 4:**
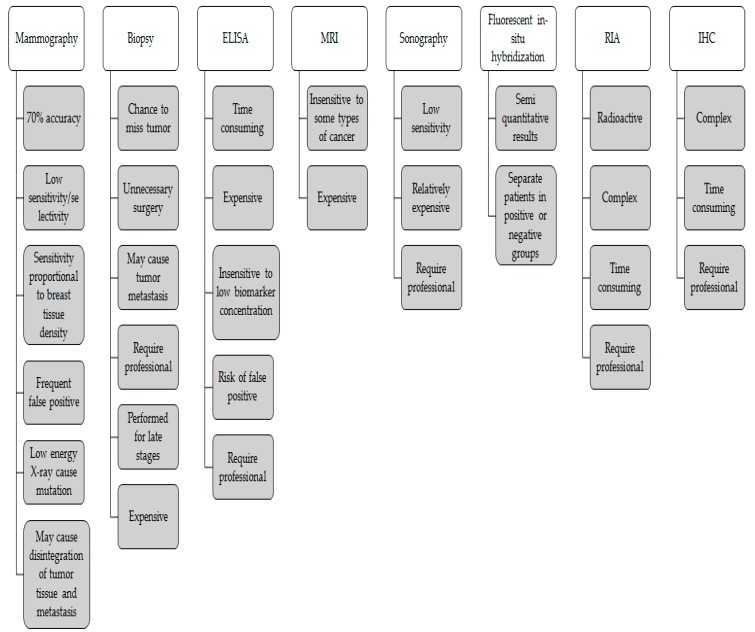
Disadvantages of the techniques used for breast cancer (BC) screening [8].

There are several screening techniques for BC their advantages and disadvantages are summarized in Figure 4.

Here, we would like to discuss the major techniques used for the early detection of BC as well as their major drawbacks.

### 4.1. Mammography 

Mammography is beyond doubt the quintessential imaging modality used today for the evaluation of BC. It is used both for screening asymptomatic BC and helping with the diagnoses. It uses X-ray to produces the images [32].

A key note to make is that mammography is plagued with false positives [33] and overdiagnosis [34,35]. A study in France showed that nearly 27% of those who undergo screening for early detection are subjected to overdiagnosis [36]. While in Lebanon, the trend of using mammography is very recent (as early as 2002) which means that not enough data has been collected for a fundamental analysis, however, early studies have shown promising results [37]. 

However, these limitations do not disapprove the life-saving benefits of mammography, with numerous studies showing the benefits of early detections with mammography in saving lives and increasing treatment options. However, and this is especially true for countries with a poor healthcare infrastructure, population-based and organized mammography screening programs are not exactly cost-effective. In these cases, awareness programs for early signs and screening by clinical breast examination are recommended [1].

### 4.2. Clinical-Breast Examination (CBE) and Breast Self-Examination (BSE)

**Figure 5:**
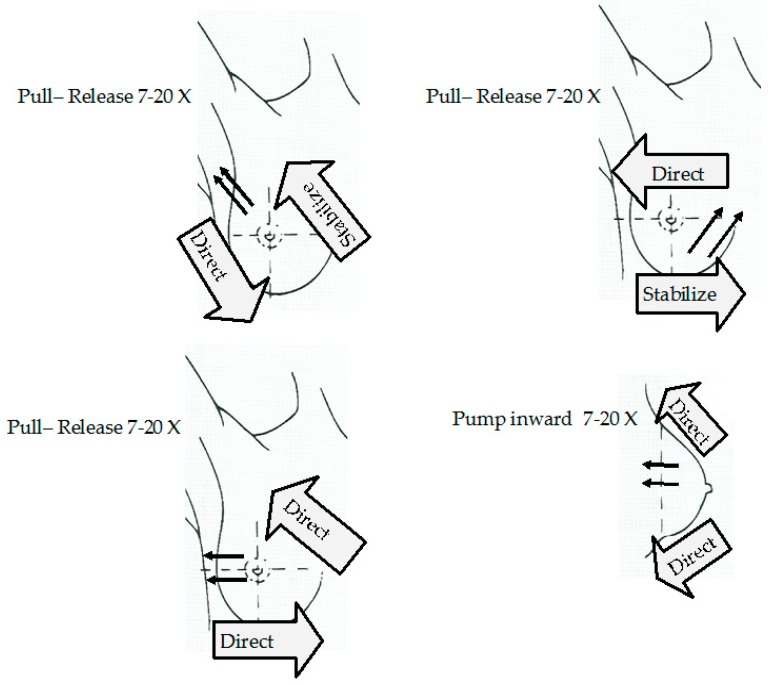
Diagrams showing how to perform a clinical breast examination (CBE) [39].

CBE is a commonly trained ambulatory care skill as part of a physical examination. it refers to physical examination of the breast by a doctor or other health practitioner. It is not meant however to replace mammography or other imagery-based screening techniques. it is used both for screening and diagnostic procedure for BC [38,39]. A model of CBE can be found in Figure 5. CBE and BSE efficiency is yet to be verified by randomized controlled trials but, under certain circumstances, they have shown benefits [6]. 

Both detection methods show almost the same in relation to tumor size of the detected lesion regardless of the location. However, the legions in the central region of the breast were often larger than those in the peripheral region [40]. 

In countries where BC diagnosis occurs at the advanced stage, screening with CBE and BSE will perhaps be useful in reducing BC mortality, although, in countries with adequate treatment and techniques, it is unlikely to be beneficial, especially with high rates of false positives and over diagnosis [6]. 

## 5. Body Fluids and the BC Biomarkers Found in Them

In 1965, the discovery of the carcinoembryonic antigen (CEA) was the first ever time cancer biomarker found in the blood stream [41]. That finding sparked the hunt to find more cancer biomarkers, in the blood at first and then from other body fluids. In this part of the review, we discuss the use of body fluids in BC biomarker detection as well as the biomarkers that have been detected.

### 5.1. Blood

The most important role of blood is maintaining the homeostasis of all cells in the body; it does so by exchanging cellular debris with nutrients acquired through the digestive and respiratory systems. This makes the blood exceptionally rich in nutrient and cellular content [42]. This property enables the blood to possess valuable information about an individual’s health status. This, by inference, makes it the perfect medium for the development of noninvasive diagnostic tools for cancer.

Despite the huge number of blood cancer biomarkers discovered, blood based diagnostic tests for the detection of asymptomatic cancer are a rarity for a few reasons:

Firstly, a lack of understanding of cancer’s molecular features and inadequate technologies inhibit the development of such tests.

Secondly, most investigations on the subject have a single improvised objective with limited resources. 

Finally, the specimens required for the discovery and validation for any given biomarker are inherently hard to acquire in the quantity and quality necessary for the said discovery and validation.

As of today, countless novel biomarkers have been discovered using the latest in quantitative analysis techniques. In addition to their roles as well as their characteristics; these biomarkers range in nature from circulating proteins, nucleic acids, metabolites, and even tumor cells. However, for many of these biomarkers, there is a lack of definitive studies for their specific application in a clinical setting, in addition to a lack of side-by-side comparison of their respective performances to determine any additive contribution [43,44]. 

### 5.2. Saliva

Saliva is a mixture of different biological fluids originating from the many salivary glands in the buccal cavity. The composition of the end-mixture (such as saliva) varies depending on a plethora of factors including the gland type it originated from, time of day, diet, age, gender, a variety of disease states, and several pharmacological agents. 

Its composition is exceptionally diverse, from respiratory mucosal secretions, serum and blood to oral wounds, bacteria, virus, fungi, epidermal and other cellular components, protein metabolites and nucleic matter, and food remains, which is why it was named as “the mirror of the body’s health” [10]. 

Regarding its use as a diagnostic medium, saliva presents several biochemical advantages over blood. For a start, its collection is safer than blood (no needle punctures), making it both noninvasive and relatively simple to collect with the capacity for repeated collection without a patient’s discomfort. In addition, salivary biomarkers have been found to be a filtrated fraction of the blood, making them a reflection of the physiological condition of the body. 

Emerging technologies that utilize saliva biomarker, particularly those based on metabolomics, have shown exceptional promise in clinical strategies for the characterization of several types of diseases, and the salivary biomarker’s capacity in the assessment of head and neck cancer has been assessed. However, with regard to BC, its usage remains mired with uncertainty due to lack of any substantial evidence for its use [45]. 

The reason is that saliva analyses are often viewed as less reliable than blood or urine analyses primarily because saliva is a difficult matrix to manage. For a start, salivary pH is influenced by flow rate, which alters analyte excretion and because analyte concentration in saliva is lower than in urine or blood, stimulated saliva is the most common course of action to acquire a sample volume sufficient enough for analysis. In addition, salivary immunoassays are also subject to matrix effects; mucins and alpha amylase in saliva have been reported to cause suppression of antibody binding in some steroid and testosterone immunoassay detection, a finding that adds a matrix removal step in many types of assays [46].

### 5.3. Others

As the carrier of blood waste, urine has been investigated for potential information on blood and kidney diseases for centuries. It is inexpensive, rich in metabolites, easy to handle, and available in large amounts in a noninvasive manner. However, the fact that these metabolites are often found in low quantities with a large inter-patient variety, limit its use [47]. 

However, several researches have been conducted to identify targets for the early detection of BC, some focusing on DNA/microRNA in urine as potential markers [48,49], while others focusing on metabolites [50] and even protein biomarkers [51,52]. The state of research for urinary biomarkers for the detection of BC is still in its infancy and no results have been forthcoming to create a substantial shift in their clinical use.

### 5.4. BC Biomarkers Found in Biological Fluids

Measurement of tumor biomarkers is held on the understanding that cancer cells differ from their normal counterparts, and that this difference is not only detectable but measurable. Current attempts at using serum tumor markers for cancer screening in the past have been in vain due to their low specificity and sensitivity at the early stages of the disease [53]. However, the usage of protein biomarkers for the prognosis and predictive behavior of BC is widely known and a large number of commercially available tests are present in that regard [54].

Normally, various biomarkers are used for various tasks, an example of this is carcinoma antigen 125 (CA125) a serum biomarker for monitoring ovarian cancer with possible use for prediction of the patient’s therapy response but this lacks the sensitivity for diagnosis. Prostate-specific antigen (PSA) is effectively used not just for early detection of but also for monitoring prostate cancer [55]. 

In a recent article by Elisa Cançado Porto-Mascarenhasa, published in 2017, a meta-analysis was done to identify all the different BC salivary biomarkers [45]. Some of the most notable results of that analysis can be found in Table 2. 

#### 5.4.1. Human Epidermal Receptor (HER) Family 

The HER family is an important family of protein biomarkers that include the epidermal growth factor receptors (EGFR or HER1 or c-erbB-1) and HER2.

EGFR is an important receptor that directly dictates the behavior of epithelial cells [58]. Their overexpression in triple negative types of BC is often an indication of a bad prognosis and ultimately worse survival odds [59]. 

HER2 also known as HER2/neu or c-erbB-2 is an important biomarker for the diagnosis of HER2-enriched types of BC, with circulating HER2 levels as predictors for the presence and progression of HER2 positive BC cell lines [60]. More recently, salivary levels of HER2 have been suggested to be used in the detection of HER2 types of BC [61]. HER2 has been a useful biomarker as prognostic indicator for decreased rates of survival of patients with a metastatic BC, and often an indicator of the tumor’s proliferation into other organs [62]. HER2 is also listed as one of the recommended biomarkers for monitoring the response to therapy for HER2-enriched BC by the European Group on Tumor Markers (EGTM) [63]. 

#### 5.4.2. CEA

CEA is a glycoprotein found in the serum of cancer patients. Its main function is the detection of metastasis of gastrointestinal cancer. The serum level of CEA does not provide a good evaluation for the metastasis of BC as large quantities of false positives have been reported [64]. 

#### 5.4.3. Mucins 1

Mucins 1(MUC-1), also known as CA 15-3, and CA 27.29, are the only types of biomarkers approved for use by the FDA for the monitoring of BC [64]. 

In healthy cells, mucins’ role is the protection, lubrication, and hydration of the external surfaces of epithelial tissue layers lining ducts and lumens in different parts of the body. Any alteration in its expression, however, has been directly linked to cancer formation and metastasis. 

CA 15-3 and CA 27.29 are antigens of the MUC-1 protein; they are specific sites on the proteins in which an assay has been developed for their detection [65]. 

Serum CA 15-3 is mainly used in the determination of BC prognosis and therapy efficacy; this clinical function is due to CA 15-3 serum levels being proportional to different stages and size of the tumor. It has also been shown that CA 15-3 in saliva is 50% higher with breast cancer patients than with the control group [57]. 

CA 27.29 has been shown to possess higher sensitivity and lower specificity than CA15-3 [62,64]. 

#### 5.4.4. Tumor Protein 53 (p53)

p53 is an important protein that has been linked to causing cellular apoptosis once it detects DNA damage. A few types of BC have been known to mutate p53 to deactivate it, other types alter the p53 apoptotic cascade by deactivating its cofactors. Clinically, p53 is mainly used to gauge the effectiveness of chemotherapy for BC due to its increase when tumor damage occurs [62,66]. Salivary levels of p53 have been shown to be 25% less in breast cancer patients than in healthy individuals [57]. 

#### 5.4.5. VEGF and EGF

Vascular endothelial growth factor (VEGF) and EGF are two salivary biomarkers that have been shown to have the highest sensitivity and selectivity among a host of biomarkers through ELISA testing [67]. In the serum however, VEGF and EGF are often used to monitor post therapy patients with metastatic BC [68]. 

#### 5.4.6. Other Proteins

A few more biomarkers are still under investigation (Cathepsin D, Cyclin E, Human epidydimal protein 4), but not enough studies have been undertaken for them to be efficiently used in a clinical setting [62]. 

#### 5.4.7. Autoantibodies

Autoantibodies are antibodies that target specific cancer biomarkers produced by the body, these antibodies have been shown to target nearly all other breast cancer protein biomarkers. These antibodies are useful precursors for early breast cancer [69]. In a recent article, salivary autoantibodies against both HER2 and MUC-1 have been shown to be useful candidates for early detection of breast cancer [70]. 

#### 5.4.8. MicroRNA

MicroRNAs or mRNA are short sequences of non-coding RNA nucleotide (21–25 base pairs long). They function as gene expression regulators at the post-transcriptional level. The current consensus is that mRNA have an important function in tumor development; tumors amplify oncogenic mRNA production while suppressing anti-tumor mRNA production, thus creating a distinct mRNA signature that can be used to identify tumor molecular subtypes while also playing a part in tumor initiation, metastasis, and drug resistance [71]. 

With regard to diagnosis, more than 133 mRNAs have been found to present abnormal expression levels in BC tissues when compared with normal breast tissues. However, discrepancies among the different reported mRNA signatures have delayed their widespread use. This is most likely due to the intrinsic heterogeneity in BC as discussed above [72].

Zhang et al., 2010, found the presence of eight different mRNA (CSTA, TPT1, IGF2BP1, GRM1, GRIK1, H6PD, MDM4, S100A8) in saliva that can be used for the detection and identification of BC [73].

## 6. Affinity Sensors (AS)

Enzyme-Linked Immunosorbent Assay (ELISA) is one of the most used standard techniques for the confirmation of the existence of specific protein biomarkers in a patient. However, it has a lot of drawbacks in comparison to modern techniques; it has low-throughput, it is expensive, and it needs a large sample size in order to acquire the desired sensitivity [22].

Recently however, Affinity Sensor (AS) use for the detection of cancer has garnered increased interest due to its excellent analytical performance and real-time measurement.

AS are devices that transform a biological interaction into a physical one [74]. Their low detection limit allows the measurement of low concentration biomarkers in in-vitro samples, thus aiding in cancer diagnosis. Furthermore, they possess the ability of simultaneous biomarker detection, reusability of biorecognition molecules, and little to no sample preparation [9]. 

After a decade development, the most prominent AS in commercial use remains the glucose amperometry devices and the lateral flow pregnancy test. However, many schemes are showing promise for future development and use.

**Figure 6:**
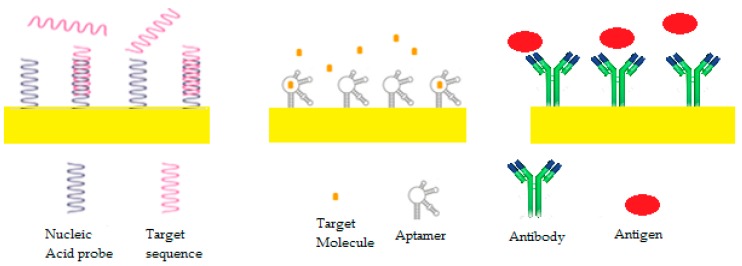
Types of biomarkers and bioreceptors used in affinity sensors (AS) design [9].

**Figure 7:**
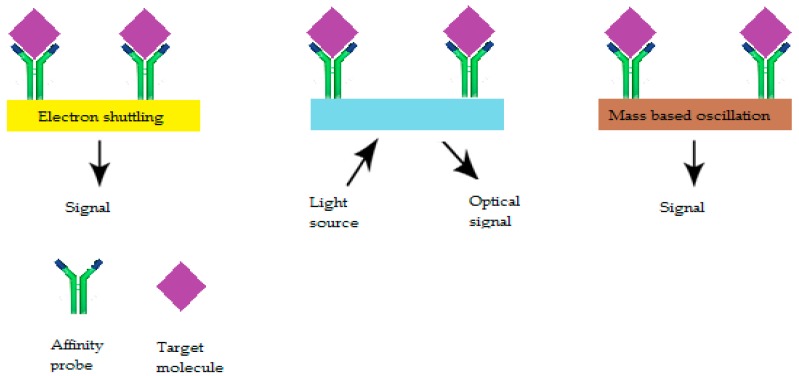
Type of biotransducers used in an AS [9].

Figure 6 and Figure 7 explain the different types of bioreceptors and which type of transduction mode can be used.

It is important to note that many assays have been developed to accommodate the many biomarkers that exist, of those we list the most notable ones:

- Direct assay: also known as label free assay, the biomarker is immobilized right on the surface where a detection antibody against the biomarker is used for sensing. Direct assay is the most preferred due to it being low cost and a simple, one step, procedure for the detection of target molecules, its main limitation is the lack of significant change during the recognition of the target [75,76].
- Capture assay ‘‘sandwich’’: A capturing antibody is first immobilized on the surface before the addition of the biomarker. Then the biomarker is sandwiched between it and an intermediate antibody before a third labeled antibody for the detection. This method is used to enhance the signal that is generated by the recognition of the target. Several materials have been used as labels or tags for labeling or even for enhancing the detection. The most notable ones are:
  ○ Nanoparticles (NP): NPs are particles that range in size from 1 nm to 100 nm, ranging in materials from inorganic to organic in nature. They have been used in AS set-ups mainly due to their optical properties which differ from normal materials through their plasmonic properties which change with any surface change providing the basis of sensing. Also, they have useful fluorescent and electrochemical properties which have been exploited in different set-ups [77].
  ○ Enzymes and proteins that can generate a fluorescence signal or an electrochemical signal through oxido-reduction reaction [78].

AS are often divided into their constitutive components, mainly the bioreceptors and the biotransducers.

### 6.1. Bioreceptors

The bioreceptors are the biological components of AS, their interaction with the target analyte is what is used to turn the biological signal into a physical one. Up until now, only four types of interactions have been used in AS development: DNA/RNA, antibody/antigen, enzyme/analyte, and aptamers/target molecule (Figure 7).

#### 6.1.1. DNA/RNA AS

Since their conception, DNA microarrays have been the most important techniques to detect DNA strands in a given solution; they are still used to detect miRNA, single nucleotide polymorphism, and other factors. However, due to their bulkiness and use of a large sample size for any given detection, the technique has evolved, and DNA based AS has become its successor [79]. 

As their name implies, DNA based AS utilizes single stranded DNA molecules to detect a targeted DNA sequence through hybridization. This in turns allows for the said hybridization to be measured through a transducer. 

Because of this property, DNA AS can be used to detect a multitude of markers such as molecular and medical diagnosis, pharmacogenomics, drug screening, food analysis, and even bioterrorism and environmental monitoring [80]. 

In BC, DNA based AS has been widely developed for the detection of BRCA1 and two genes as well as some carcinomic miRNA such as miR-21 [8].

#### 6.1.2. Peptide Nucleic Acid (PNA) AS

PNA is an artificial nucleic acid analogue. Its backbone is comprised of N-(2-aminoethyl)glycine motifs linked via peptide bonds. When coupled with DNA or RNA, the uncharged backbone of the couple PNA/nucleic acid makes the complex more stable than its corresponding nucleic coupling—it exhibits chemical and thermal stability in conditions that degrade other nucleic acids. Their insensitivity to ionic strength and pH changes, resistance to enzymatic cleavage and higher levels of selectivity toward their target sequence makes them the perfect alternative to native DNA base pairs. 

Despite not being used for the detection of BC specific biomarkers, PNA has been successfully used for the detection of mutated EGFR genes in lung cancer cell-line, showing its potential for further use as biomimetic molecules [81].

#### 6.1.3. Antigen/Antibody 

An AS that utilizes the binding of an antigen to an antibody to produce a signal, is often called an immunosensor. Immunosensors are powerful analytical tools that are mainly used for the detection of proteins and are developed to detect biological markers for diagnostic and clinical purposes. Because of their ease to work with, most of the assays that have been mentioned in the previous section use this type of sensor in some form or another [82]. 

In principle, immunosensors utilize immunoglobins (also called antibodies or Ab), a protein that is secreted by the immune system’s B cells that targets an intruder protein via humoral response. These immunoglobins have a high specificity toward their targets, which they bind strongly to, in getting rid of them. The target proteins (also called antigen or Ag) are then guided to a macrophage to be degraded. 

The binding of the Ab/Ag is mediated through the following reaction:

(1) Ag+Ab↔AgAb

It follows the equilibrium equation bellow:

(2) $$Ka=\frac{\left[AgAb\right]}{\left[Ag\right]\left[Ab\right]}$$

with Ka being the affinity constant of the said binding [83,84]. 

This property of Ab Ag binding has been utilized in many assays before; the most famous of which is the sandwich ELISA (Enzyme Linked Immuno-Sorbent Assays) test [42].

#### 6.1.4. Enzyme Biosensors

The most prominent enzymatic biosensor to date has been the use for the detection of diabetes mellitus, this makes the enzyme biosensor the only biosensor to ever become part of the mainstream. A key feature of these enzymes is their use of Glucose Oxidase (GOx) as a reducing agent in order to change the electrical composition of the solution through the following equation:

(3) $$Glucose+O_{2}\to Gluconolactone+H_{2}O_{2}$$

Since the nature of the reaction is an oxidation reaction, the easiest transducers to be used for the development of these biosensor are the electrochemical ones with the amperometry biosensors taking center stage [85,86]. 

Another area where the enzyme biosensor is being used is in the detection of pesticides. Their ability to inhibit enzymes has been employed in designing several biosensors tailored for their detection [87]. In BC however, enzymes are not used for any particular detection of a biomarker, but, they are used in tandem with a specific bioreceptor as a signal amplification strategy. 

#### 6.1.5. Aptameric Biosensors

Aptamers are a form of in-vitro nucleic acid ligands, they are artificially created from virtual libraries through a process called systematic evolution of ligands by exponential enrichments (SELEX). 

Their self-annealing properties give them an advantage over normal base pairings such as DNA/RNA in that they can form specific 3D structures granting them high selectivity and binding affinity comparable to or even greater than those of their corresponding Ab.

However, and unlike Ab, the selectivity and affinity of aptamers can be tailored to the specific needs of the selection process; this is a key advantage over traditional bioreceptors in that aptamers can be designed for a specific target (DNA/RNA, proteins, small molecules etc.) with greater stability and without the need for experimental animals.

When it comes to BC detection, aptamers have been used as bioreceptors for AS, to probes for capturing the target molecule and/or BC cells, they have even been coupled with IHC to yield greater sensitivity and selectivity when assessing cell lines [88,89].

#### 6.1.6. Molecularly Imprinted Polymers (MIPs)

MIPs are affinity polymers created for various targets of analytical interest (inorganic and organic molecules, drugs, nucleic acids, proteins). They are used as an alternative to natural biomolecules because of their stability, specificity, and general low cost. MIPs are created through the formation of a complex between a target molecule (template) and functional monomers through covalent or non-covalent interactions, this step is followed by the polymerization of the monomers to form a cast-like shell. The final step is the removal of the target molecule leaving behind binding sites on the polymer that have both the correct shape and orientation of functional groups similar to those on biomolecules which are then used for selective recognition of the template.

Several limitations exist that limit the widespread use of MIPs, one such limitation is the difficulty to integrate the polymer onto a transducer, though several advancements have been made in that regard, such as electro-polymerization. More work still needs to be made in order to enhance the binding kinetics, decrease the analysis time, and increase the number of binding sites by removing more of the templates from its surface [76].

### 6.2. Biotransducers

Biotransducers can be classified into three main classes: Electrochemical, Optical, and Mechanical. A list of all biosensors for the detection of protein BC biomarkers can be found in Table 3 below.

#### 6.2.1. Electrochemical AS

With low cost, versatility, and sensitivity, Electrochemical AS are often a favored choice in AS fabrication. These techniques require small sample volumes and are a good choice for the development of portable devices. They are often created inside an electrochemical cell.

There are three methods in which electrochemical sensing occurs, Conductometry, Voltammetry, and Amperometry in both static and dynamic modes, and Electrochemical Impedance Spectroscopy (EIS) [84].

● Conductometric AS

Conductometric-based detection technique is the measurement of the movement of ions inside a liquid medium upon applying a current to that medium through an interdigitated electrode. Conductance is influenced by several factors such as temperature, Faradaic processes, double-layer charging, and concentration polarization. This means that there are two disadvantages to this method: the first is that using differential methods with internal controls is a must. The second is that the ionic species production from the reaction must be important enough to change the total ionic strength for reliable measurements to occur, which means that any change in the ionic strength of the medium can cause interference in the measurement. With lower sensitivity than other electrochemical techniques, conductometry is often used only for enzymatic reactions, limiting its use to environmental monitoring, in particular, gas sensors used in alarms and control appliances [133]. 

Several AS for the detection of BC were developed using conductometry, they can be found in Table 3 above.

● Field effect transistors (FET) based sensors

The ability to directly convert specific biological interactions into an electrical signal is a specific characteristic of FET sensors. They work by modulating the charge of an electrical field through a semiconductor material.

**Figure 8:**
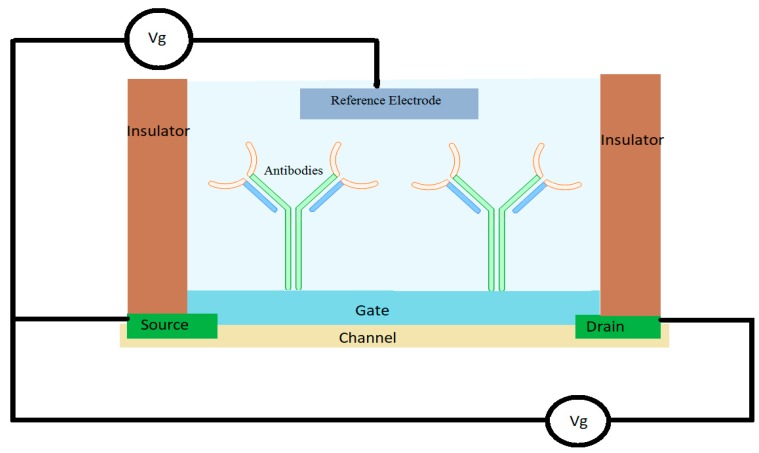
A schematic for a field effect transistor (FET)-based affinity based sensor for antibody–antigen interaction.

The usual FET arrangement contains a semiconductor channel linked to a source electrode and a drain electrode, coupled in a capacitive manner with a third electrode, also known as the gate contact, through a thin dielectric layer (normally SiO_2_) which modulates the electrical current flowing through the system. A schematic can be found in Figure 8.

The FET devices usually detect by plotting the current on the source-drain electrode as a function of the gate voltage at a constant source-drain voltage. The type of semiconductor (p-type or n-type) dictates the behavior of the current of the device.

FET based AS are simple to use, easy to produce with a low operation cost, portable, and possess high sensitivity (in the range of femtomolar under ideal conditions) while only using small volumes in real time [134].

Examples of FET-based AS for the detection of BC can be found in Table 3 above.

● Voltammetry and potentiometry

Voltammetry is the most widely used electrochemical method in dynamic conditions. It focuses on studying the current responses under different conditions. Commonly, voltammetry is designed by the relationship of voltage in volts, current in amperes, time in seconds for a three electrode system: working, counter, and reference electrodes.

Voltammetry techniques work in a similar fashion in that they measure either the static potential at the electrode (Static mode) or measure the resulting potential from an applied current on the electrode (dynamic mode). On that principle, many voltammetry techniques evolved, all of which focus on the current response (cyclic voltammetry, differential pulse voltammetry, square wave voltammetry, and amperometry).

Voltammetry can be carried out in motionless solutions, under stirring, and in vibrating solutions as well where the mass transport to the working electrode is greatly improved with stirring, yielding a better detection limit. Thus, several methods that use this property have been used for improved sensitivity, such as flow injection analysis (FIA), sequential injection analysis (SIA), or batch injection analysis (BIA) [84].

For the detection of BC protein biomarkers, voltammetry is actually the most popular method of electrochemical detection due to its versatility for both electrical flexibility and experimental setup. Table 3 highlights several of the AS developed with voltammetric detection techniques. Examples of voltammetric AS can be found in Table 3.

● Electrochemical Impedance Spectroscopy (EIS)

EIS is a potent characterization technique that is commonly used in differing fields ranging from energy to medicine. EIS is appealing for two main reasons. First, EIS data is employed to acquire the physical and microstructural properties of the electrode. Second, an EIS experiment is relatively simple to set up and implement. 

The EIS principle is the opposition force to an electrical current in a circuit. It is measured in ohms the same as resistance, although resistance is in fact impedance with a zero phase angle, such as a DC circuit. However, in the majority of cases, the phase angle is not equal to zero due to capacitive and inductive effects that are observed at every frequency, which is why a more general concept that takes frequency into consideration is used. EIS as a concept becomes a quantitative representation of these opposition forces to electrical current with varying frequencies (AC).

One method using EIS in a biological experiment is to biofunctionalize a bioreceptor on an electrode surface that will attract the target analyte, affecting the conductivity of the system by either blocking the surface or a ‘molecularly wired’ admittance mechanism.

When this strategy is used, EIS measurements are done in a blank buffer solution or a known redox probe (example: potassium ferricyanide) after the biorecognition of the target analyte has occurred on the electrode surface.

**Figure 9:**
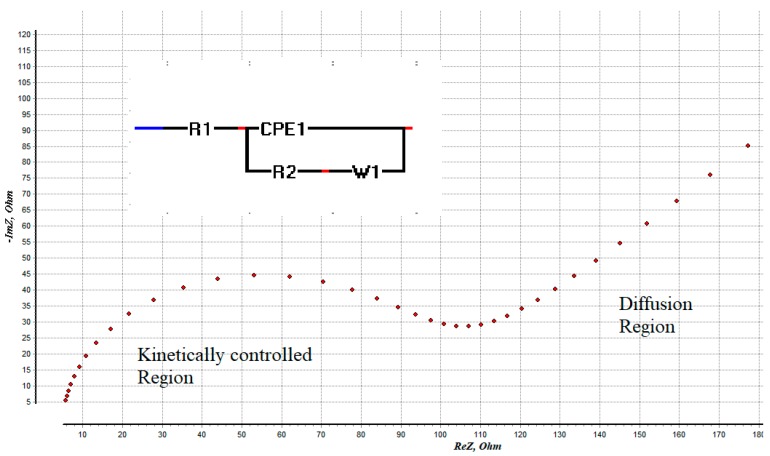
Representation of a Nyquist plot with its equivalent circuit and its related components.

What follows is then running the AC current through the electrode at a varying set of frequencies and the potentiostat record the resulting parameters and a software convert them into an impedance value with a real and imaginary component. The data is then presented in a variety of ways, for example, real and imaginary impedance components are plotted against one another in Nyquist plots which can be found in Figure 9 [135]

EIS spectra are commonly analyzed using equivalent circuits, where the operator uses his knowledge to find a circuit with a finite set of elementary (resistors, capacitors, and inductors) and generalizes an electrical circuit that can match the data. Because of their simplicity, equivalent circuits are especially attractive. One downside of this however, is in the interpretation of the results. In many practical cases, more than one model can be fitted to the experimental data equally well. These uncertainties can be additionally aggravated when some parameters of the models are not determined with adequate accuracy [136].

Many BC AS use EIS for the detection of biomarkers, some of them can be found in Table 3.

#### 6.2.2. Optical AS

As its name implies, optical AS use light properties to create their signal, many strategies were developed to employ light as a means of signal transducers.

● Surface plasmon resonance (SPR)

SPR is a phenomenon that occurs on the surface of a conducting material, usually metal, sandwiched between two reflective media also known as a dielectric, often glass or liquid. When photons from a polarized light source hit the metal at a well-defined angle, part of the photon’s energy couples with the metal’s surface and causes the electrons in the metal to vibrate. This movement of electrons is called plasmon which propagates through the metal surface, creating an electric field of 300 nm of range.

**Figure 10:**
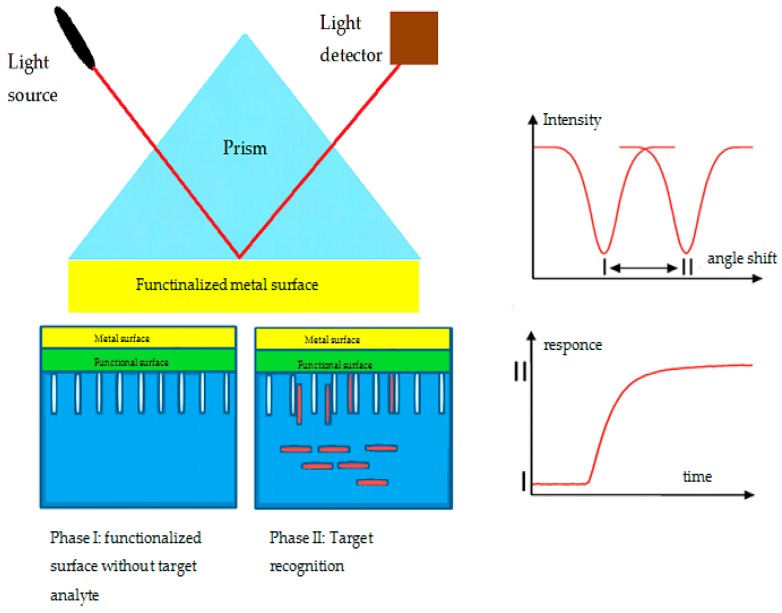
Schematic of surface plasmon resonance (SPR) biosensor design (**left**) showing the response functioning and the detection response from recognition of a biomarkers (**right**) [137].

Because of this field, two changes occur to the SPR light source as it is reflected from the surface of the metal. The defined SPR angle is reliant on the refractive index of the material near the metal surface. Therefore, when the reflective index of the sensing medium is altered after immobilization of the target molecule, plasmon cannot be formed. This means that measuring the change in the reflected light becomes a form of detection. In addition, the amount of target analyte that was immobilized on the surface can also be quantified by measuring the reflected light intensity as well as tracking the resonance angle shift [137,138]. The SPR interface can operate at a much smaller scale than the incident wavelength. All of this has allowed SPR to breathe new life into optoelectronics and nanophotonic point-of-care devices. A schematic of a functional SPR biosensor can be found in Figure 10.

Despite its several advantages, SPR suffers from a few setbacks, and one key challenge is that the close proximities of the metal/dielectric interface of a sensing platform cause the changes in the dielectric properties of the said interface to be negligible, thus drowning the detection signal. Several advances have been developed in order to circumvent this, from the various schemes to enhance localized SPR signals, to using chiral properties coupled with SPR, magneto-optical effects, and even developing quantum SPR [139].

● Fluorescence biosensors

Fluorescence biosensors are currently the most widely used of the methods in the imaging of biological and biomolecular processes owing to their high sensitivity and selectivity, sufficient temporal and spatial resolution, and low cost [140].

Currently, two major classes of these biosensors are in use: 

The Genetically-encoded single-chain FRET (Förster Resonance Energy Transfer) biosensors are single-chain FRET biosensors, also called Kinase Activity Reporters (KARs), that express a pair of genetically-encoded autofluorescent proteins (AFPs) bordering a substrate sequence and a phospho-amino acid-binding domain (PAABD) joined by a linker [141].

Non-genetic fluorescent biosensors use a protein or polymer scaffold conjugated with an environmentally responsive fluorophore. 

The advantage of genetically encoded AFPs biosensors is their ability to be basically expressed in all types of cells, permitting simple visualization of intracellular target molecules. 

In contrast, non-genetic fluorescent biosensors require an invasive technique to enter the cell membrane, such as electroporation, lipofection, microinjection, and tagging arginine-rich sequences. However, this type of biosensor could be more strategic to use in several cases. The smaller fluorophore causes less perturbation to the original receptor protein properties, in addition to greater control of the quantity of biosensor injected into the cell which causes minimal interference to the molecular geometry of the analyte cell [140,141].

● Electrochemiluminescence

Electrochemiluminescence (ECL) is a method that combines optical and electrochemical techniques. ECL uses light quantas emitted by the deactivation of excited molecules of electroluminophores. These emissions then change the experimental medium through: electrochemical, chemical, and photonic interactions.

ECL has many characteristics in comparison to other type of detection methods, it does not use radioisotopes, it does not use photonic excitation which gives it less background noise than other optical techniques, it has lower limits of detection, rapid measurements, and greater electrode surface control than other techniques, it can also be designed for multi-meter analysis [142].

Examples of optical AS used for the detection of BC biomarkers are found in Table 3.

#### 6.2.3. Acoustic Wave Sensor

During the late 1970s, the increased demand for commercial products in the telecommunication area became the foundation for mass-producing devices that utilized electroacoustic technologies. Then, these devices became suitable for electronic applications such as electronic data processing and high-frequency technology. These devices, also known as surface acoustic wave (SAW) devices, utilized frequencies in the range of 100 MHz to a few GHz that are strongly confined at the device’s surface (usually made of piezoelectric crystals) within the above mentioned range regardless of its thickness. This property made the waves very sensitive to any change on the surface, including mass loading, viscosity, and conductivity change. In 1979, this property led to the creation of the first SAW device for gas detection [143,144].

**Figure 11:**
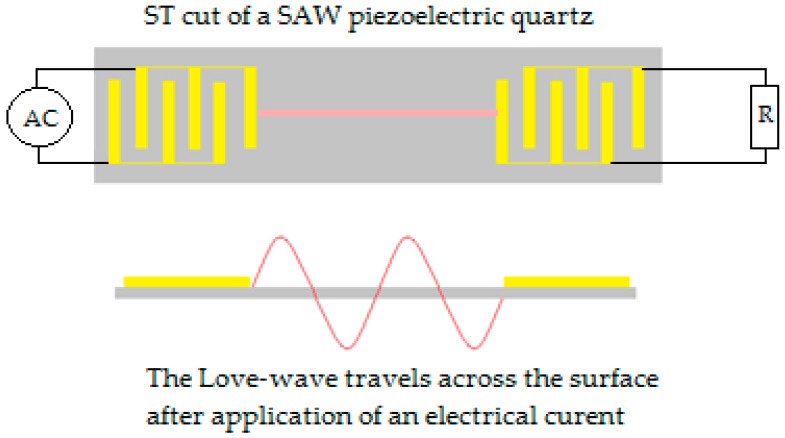
Graphic representation of surface acoustic wave (SAW) biosensors with an ST-cut.

However, SAW devices were ill-suited for use in aqueous solutions as the surrounding liquid often dispersed the signal. This challenge was overcome by creating different cuts of the piezoelectric quartz materials in order to obtain different properties of the signal such as the ST-cut used for SAW detection. An example of the cut can be seen in Figure 11. 

The energy of a SAW biosensor signal is confined close to the planar surface of a solid medium. The utility of such confinement is an increased sensitivity to surface adsorption. Biosensors that use SAW are often made of interdigitated metal electrodes molded on a piezoelectric substrate which can be made into a thin film on silicon. The piezoelectric substrate is then excited with the acoustic wave and the detection is the responding change of the wave as seen in Figure 11; the SAW is most efficient when its wavelength is equal to the spacing between the transducer fingers [145].

Love wave is an application of SAW sensors that uses a specific type of wave called a horizontally polarized shear wave, which is a form of low velocity wave that minimizes the losses in the acoustic wave after it enters into the bulk of the substrate or if the substrate is immersed in a liquid. In addition, this reduction in wave weakening can be attained if the layer is fine-tuned to an optimal thickness. In this case, an optimal thickness increases the sensitivity of the biosensor towards any change in its surface’s physical properties such as mass load [144].

An example of acoustic AS used for BC biomarkers can be found in Table 3.

## 7. Conclusions

BC screening, despite the many drawbacks related to the current techniques used, has had a significant impact on the fight against the disease. It is no longer a question whether BC screening is something that needs to be done, it is however a question on how to increase its efficiency. While imaging techniques still have room to evolve, serum BC biomarkers have already been shownto have their limitations.

In contrast, saliva is one of the most virgin body fluids in terms of sensor development, as limitations caused by the salivary matrixes have discouraged researchers in the development of practical point-of-care devices for the detection of biomarkers in them. 

Despite these limitations, the development of salivary BC biomarkers has made great strides in recent years with actual targets already identified, and the development of devices that are inexpensive, easy to use, and with sufficient sensitivity already underway. Still, the lack of substantial research on this subject further limits the clinical use of affinity sensors for salivary BC biomarker detection.

We are fast approaching a critical point in the detection of salivary BC biomarkers as many of the sensors discussed in this article are able to detect at the levels of these biomarkers. It is only a matter of time before the right technology arrives to upend the current status quo.

## Figures and Tables

**Table 1:** Molecular classification of breast cancer (BC) and the respective prognosis [19].

Molecular Subtypes	ER *	PR **	HER2 ***	Histological Grade	Basal Marker	Proliferation Cluster	P53 Mutation	Outcome
Luminal A	+	+	−	Low	−	Low	Low	Good
Luminal B	±	±	−/+	High	−	High	Intermediate	Intermediate
Basal like	−	−	−	High	+	High	High	Poor
HER+/ER−	−	−/+	−/+	High	±	High	High	Poor
Molecular apocrine	−	−	±	Intermediate/High	±	High	Intermediate/High	Poor
Claudine-low	−	−	−	High	High	High	High	Intermediate

* ER: Estrogen Receptor, ** PR: Progesterone receptor, *** HER2: Human Epidermal Receptor 2.

**Table 2:** Concentration of some notable salivary breast cancer biomarker proteins [56,57].

	c-erbB-2		CA 15-3		p53		EGFR ***	
Subject Status	C * U/mg	TSP ** mg/mL	C U/mg	TSP mg/mL	C pmol/mg	TSP mg/mL	C fmol/mg	TSP mg/mL
Control	71.13 ± 66.07	1.48 ± 60.09	2.27 ± 1.54	1.25 ± 0.82	177.1 ± 61.3	1.25 ± 0.82	1.03 ± 0.69	1.25 ± 0.82
Benign	63.75 ± 67.00	1.58 ± 0.08	2.22 ± 1.95	1.44 ± 0.92	180.7 ± 70.7	1.44 ± 0.92	0.37 ± 0.31	1.44 ± 0.92
Malignant	143.58 ± 611.53	1.67 ± 60.10	5.26 ± 4.12	1.71 ± 0.79	134.6 ± 63.8	1.71 ± 0.79	0.92 ± 0.80	1.71 ± 0.79

* C: protein concentration. ** TSP: total protein concentration. *** EGFR: epidermal growth factor receptor.

**Table 3:** List of affinity sensors (AS) for the detection of BC protein biomarkers.

Protein	Electrode Type	Essay Type	Amplification	Detection Methods	Limit of Detection	Medium of Detection	Reference
CA15-3 ^9^	Screen-printed carbon electrode, functionalyzed with polypyrrole	Sandwich assay	Magnetic beads	Amperometric	0.02 U/mL	PBS ^1^	[90]
CA15-3	Gold electrode	label free assay	Ferrocenecarboxylic doped nanoparticles	Amperometric	0.64 U/mL	PBS, human serum	[91]
CA15-3	Gold/Graphene glassy carbon electrode	Sandwich assay	HRP ^2^-encapsulated liposomes	Differential pulse voltammetry	5 µU/mL	PBS, human serum	[92]
CA15-3	N-doped graphene sheets on glassy carbon electrode	label free assay	-	Differential pulse voltammetry	0.012 U/mL	PBS, human serum	[93]
CA15-3	Graphene oxide modified gold electrode	Sandwich assay	Ab ^3^/ferritin/multiwall carbon nanotube	Differential pulse voltammetry	0.009 U/mL	PBS, human serum	[94]
CA15-3	MIPs ^4^/SPE ^5^	Direct assay	Toluidine blue	Differential pulse voltammetry	0.1 U/mL	PBS/artificial human serum	[95]
CA15-3	MIPs/gold SPE	Direct assay	Hexacyanoferrate (II/III)	Differential pulse voltammetry/ EIS ^6^	1.5 U/mL	PBS/human serum	[96]
CA15-3, CA125 ^10^, CEA ^11^	screen-printed carbon electrode	Sandwich assay	platinum nanoparticles	Conductometry	0.002 U/mL, 0.001 U/mL and 7.0 pg/mL for CA125, CA153 and CEA, respectively	PBS, human serum	[97]
HER2 ^12^+ CA15-3	Screen printed carbon electrode	label free assay	gold nanoparticles	Linear Sweep voltammetry	5.0 U/mL for CA 15-3 and 2.9 ng/mLfor HER2	PBS	[80]
CA15-3	Glassy carbon electrode modified with gold nanoparticles	Sandwich assay	Palladium nanocage captured Ru(II) luminophore	ECL ^7^	0.003 U/mL	PBS, Human serum	[98]
CA15-3	graphene oxide modified screen printed carbon electrode	Sandwich assay	Modified magnetic nanoparticles	ECL	2.8 × 10^−4^ U/mL	PBS, Human serum	[99]
CA15-3	Gold electrode	Sandwich assay	2 types of Lectin modified fluoromicrobeads, Sambucus nigra agglutinin and peanut agglutinin lectins	Fluorescence	1.2 U/mL for PNA and 0.4 U/mL for SNA	PBS	[100]
CA15-3	Gold nanorods	Direct assay		Plasmonic	0.1 nM	Human serum	[101]
CA15-3	Glassy carbon electrode	Direct assay	3D DNA nanomachine probes using Protein-Aptamer binding complex, a mimic peroxidase	ECL	0.62 fg/mL	PBS, Human serum	[102]
HER2	screen printed electrode	Sandwich assay	HRP linked Ab	Cyclic Voltammetry	4 ng/mL	PBS, human plasma	[103]
HER2	screen printed electrode	Sandwich assay	Magnetic beads modified with enzymes, affibody were used instead of antibody	Differential pulse voltammetry	1.8 ng/mL	PBS, human serum	[104]
HER2	Gold electrode	Sandwich assay	Aptamer, DNA primer	Amperometric	1 pg/mL	PBS, human plasma	[105]
HER2	MIPs/gold SPE	Label-free assay	Ferro-ferricyanide	Differential pulse voltammetry	1.6 ng/L	PBS, human serum	[106]
HER2	Aptamer hybridized on ferrocene labeled DNA gold nanoparticles	Sandwich assay	horse radish peroxidase labeled DNA gold nanoparticles	Conductometry	4.9 ng/mL	PBS, human serum	[107]
HER2	Gold electrode	Sandwich assay	Aptamer, DNA primer	Conductometry	-	PBS, human serum	[108]
HER2	screen printed graphite	label free assay	gold nanoparticles	EIS	6.0 μg/L	PBS, human serum	[109]
HER2	Gold electrode	label free assay	DNA aptamer	EIS	1 pM	PBS, Diluted human serum	[110]
HER2	36°YX-LiTaO _3_ device with gold transducers	label free assay	Neutravidin and Protein A	Surface acoustic wave	2 ng/mL	PBS	[111]
EGFR ^13^	Gold electrode	label free assay	ferrocene bead coupled with peptide	EIS	0.37 ng/mL	PBS, Diluted human serum	[112]
EGFR	Gold electrode	label free assay	gold nanoparticles	EIS	0.34 pg/mL in PBS and 0.88 pg/mL in human plasma	PBS, human plasma	[77]
EGFR	screen printed electrode	Sandwich assay	Ferro oxide/Chitosan/Gold nanoparticles	differential pulse voltammetry	-	PBS, human plasma	[113]
EGFR	Zinc-Oxide	Direct assay	-	FET-based sensor	10 fM	Goat serum	[114]
EGFR + HER2	Graphene encapsulated nanoparticles	Direct assay	-	FET ^8^-based sensor	1 pM for HER2 and 100 pM for EGFR	PBS	[115]
EGFR	Gold electrode	label free assay	-	Cyclic Voltammetry	1 pg/mL	PBS	[116]
CEA	screen printed electrode	label free assay	Lectin	Chronoamperometry	0.03 ng/mL	PBS, human plasma	[117]
CEA	Gold electrode	label free assay	gold nanoparticles	Differential pulse voltammetry	0.015 fg/mL	PBS, human plasma	[118]
CEA	gold electrode	Sandwich assay	gold nanoparticles	Square wave Voltammetry	0.2 ng/mL	PBS, human plasma	[119]
CEA	screen printed carbon electrode	Sandwich assay	ferrocene carboxylic acid liposome	Square wave Voltammetry	1 pg/mL	PBS, human plasma, human saliva	[120]
CEA	Graphene electrode	Sandwich assay	Gold nanorods modified with HRP and hairpin-oligonucleotide	Conductometry	1.5 pg/mL	PBS, human serum	[121]
CEA	Graphene electrode	label free assay	gold nanoparticles	EIS	0.06 ng/mL	PBS, human plasma	[122]
CEA	Aptamer nanocluster pair	Direct assay	-	Fluorescence	0.1 ng/mL	3-(N-Morpholino) propanesulfonic acid buffer, diluted human serum	[123]
CEA	Palladium-converting nanoparticles	Direct assay	-	resonance energy transfer	2 pg/mL	Tris-HCl buffer, diluted human serum	[124]
CEA	ST 90°-X quartz	Sandwich assay	Gold nanoparticles	Love wave	30 pg/mL	PBS	[125]
VEGF ^14^ +PSA ^15^	Gold electrode	label free assay	aptamer	Square wave Voltammetry	1.1 ng/mL	PBS, cell lysate	[126]
VEGF +PSA	Gold electrode modified with graphene oxide/ssDNA	label free assay	Poly-L-lactide nanoparticles	Differential pulse voltammetry	50 pg/mL	PBS, human plasma	[127]
VEGF	Gold electrode	label free assay	Magnetic graphene oxide	Differential pulse voltammetry	31.25 pg/mL	PBS, Human plasma	[128]
VEGF	Glass carbon electrode modified aptamer	Sandwich assay	Gold platinum nanocluster	Amperometric	4.6 pmol/L	PBS	[129]
VEGF	Gold electrode	Sandwich assay	magnetic beads	EIS	401 pg/mL	Diluted human serum	[130]
p53 ^16^		Sandwich assay for multiple detection	Gold nanorod, enzyme label	Square wave Voltammetry	5 pM	PBS	[131]
p53	Glassy carbon electrode	Sandwich assay	Streptavidin modified gold nanoparticles	ECL	22.8 fM	PBS, cell lysate	[132]

^1^ PBS: phosphate buffer saline. ^2^ HRP: Horseradish peroxidase. ^3^ Ab: antibody. ^4^ MIPs: Molecularly Imprinted Polymers. ^5^ SPE: screen printed electrode. ^6^ EIS: electrochemical impedance spectroscopy. ^7^ ECL: electrochemiluminescence. ^8^ FET: field effect transistor. ^9^ CA15-3: cancer antigen 15-3. ^10^ CA125: cancer antigen 125. ^11^ CEA: carcinoembryonic antigen. ^12^ HER2: human epidermal receptor 2. ^13^ EGFR: epidermal growth factor receptor. ^14^ VEGF: vascular endothelial growth factor. ^15^ PSA: prostate specific antigen. ^16^ p53: protein 53.
